# The relationship between biological and psychosocial risk factors and resting‐state functional connectivity in 2‐month‐old Bangladeshi infants: A feasibility and pilot study

**DOI:** 10.1111/desc.12841

**Published:** 2019-05-29

**Authors:** Ted K. Turesky, Sarah K.G. Jensen, Xi Yu, Swapna Kumar, Yingying Wang, Danielle D. Sliva, Borjan Gagoski, Joseph Sanfilippo, Lilla Zöllei, Emma Boyd, Rashidul Haque, Shahria Hafiz Kakon, Nazrul Islam, William A. Petri, Charles A. Nelson, Nadine Gaab

**Affiliations:** ^1^ Laboratories of Cognitive Neuroscience, Division of Developmental Medicine, Department of Medicine Boston Children’s Hospital Boston Massachusetts; ^2^ Harvard Medical School Boston Massachusetts; ^3^ College of Education and Human Sciences University of Nebraska‐Lincoln Lincoln Nebraska; ^4^ Department of Neuroscience Brown University Providence Rhode Island; ^5^ A.A. Martinos Center for Biomedical Imaging Massachusetts General Hospital Boston Massachusetts; ^6^ The International Centre for Diarrhoeal Disease Research Dhaka Bangladesh; ^7^ National Institute of Neurosciences & Hospital Dhaka Bangladesh; ^8^ Division of Infectious Diseases and International Health, Department of Medicine, School of Medicine University of Virginia Charlottesville Virginia; ^9^ Harvard Graduate School of Education Cambridge Massachusetts

## Abstract

Childhood poverty has been associated with structural and functional alterations in the developing brain. However, poverty does not alter brain development directly, but acts through associated biological or psychosocial risk factors (e.g. malnutrition, family conflict). Yet few studies have investigated risk factors in the context of infant neurodevelopment, and none have done so in low‐resource settings such as Bangladesh, where children are exposed to multiple, severe biological and psychosocial hazards. In this feasibility and pilot study, usable resting‐state fMRI data were acquired in infants from extremely poor (*n* = 16) and (relatively) more affluent (*n* = 16) families in Dhaka, Bangladesh. Whole‐brain intrinsic functional connectivity (iFC) was estimated using bilateral seeds in the amygdala, where iFC has shown susceptibility to early life stress, and in sensory areas, which have exhibited less susceptibility to early life hazards. Biological and psychosocial risk factors were examined for associations with iFC. Three resting‐state networks were identified in within‐group brain maps: medial temporal/striatal, visual, and auditory networks. Infants from extremely poor families compared with those from more affluent families exhibited greater (i.e. less negative) iFC in precuneus for amygdala seeds; however, no group differences in iFC were observed for sensory area seeds. Height‐for‐age, a proxy for malnutrition/infection, was not associated with amygdala/precuneus iFC, whereas prenatal family conflict was positively correlated. Findings suggest that it is feasible to conduct infant fMRI studies in low‐resource settings. Challenges and practical steps for successful implementations are discussed.

**Highlights**

- This pilot study, conducted in urban Bangladesh, is the first functional MRI study of infants in a low‐resource country.
- Two‐month‐old infants from extremely impoverished families exhibited less negative intrinsic functional connectivity (iFC) between amygdala and precuneus compared with infants from more affluent families.
- Prenatal family conflict positively correlated with amygdala/precuneus iFC in preliminary analyses, but limitations in sample size and data quality necessitate caution when drawing inferences.

## 1 INTRODUCTION

According to UNICEF, 19.5% of the world's children live in poverty, and most of these are in Sub‐Saharan Africa (51.7%) and South Asia (35.7%). Childhood poverty has been associated with lower performance on memory, language, and cognitive control tasks (Dean, Schilbach, & Schofield, 2018; Farah et al., 2006), diminished academic achievement (Carvalho, 2012; Luby, 2015), and greater rates of future mental health symptoms (Farah, 2017; Lund et al., 2010). Specific components of socioeconomic status (SES), such as low income and low maternal education, have also been linked to brain development (Hair, Hanson, Wolfe, & Pollak, 2015; Luby et al., 2013), which in turn may drive delays and deficits in cognitive outcomes (Noble, Houston, Kan, & Sowell, 2012).

Research linking childhood poverty to neurocognitive development has spurred efforts to identify structural and functional neural correlates of family income‐to‐needs (i.e. household family income divided by number of household members). In infants, a positive association has been observed between income‐to‐needs (or another metric of income) and frontal and parietal lobes (Hanson et al., 2013), cortical (Betancourt et al., 2016) and total (Hanson et al., 2013) gray matter volumes, and faster rates of growth in these regions (Hanson et al., 2013). However, specific brain regions were not investigated in these studies (for a review, please see Hurt & Betancourt, 2016). Studies of poverty and its association with variations in brain structure in older children (study‐average ages: 9.8–12 years) have reported positive associations between income‐to‐needs (or income in general) and volumetric properties of subcortical structures, including the hippocampus (Hair et al., 2015; Hanson, Chandra, Wolfe, & Pollak, 2011; Luby et al., 2013; Noble et al., 2012) and amygdala (Luby et al., 2013), such that these brain areas exhibited reduced volume in children from poorer families compared with children from more affluent families. Based on these findings, a general model has been proposed in which specific components of SES engender stress, which in turn alters properties of various brain regions, including the amygdala (Noble et al., 2012), a region critically tied to the stress response hypothalamic‐pituitary‐adrenal (HPA) axis (Lupien, McEwen, Gunnar, & Heim, 2009). More recently, resting‐state fMRI was used to examine the relationship between income‐to‐needs and intrinsic functional connectivity (iFC) between the amygdala and other brain areas in children (mean age: 9.9 years). Negative correlations were observed between income‐to‐needs and left amygdala/right precuneus iFC and right amygdala/left superior parietal cortex iFC (Barch et al., 2016), supporting findings from retrospective studies of adults raised in poverty in the U.S., which showed a similar relationship between income‐to‐needs and altered amygdala iFC (Javanbakht et al., 2015; Kim et al., 2013).

Importantly, poverty does not act on the brain directly, but likely does so indirectly through adverse biological and/or psychosocial risk factors (e.g. malnutrition, infections, low maternal education, and family conflict) experienced early in life (Jensen, Berens, & Nelson, 2017). To date, a few studies have attempted to identify risk factors that mediate the relationship between income‐to‐needs (a continuous variable that reflects family income per household member) and associated brain alterations (Luby et al., 2013; Noble et al., 2012), but no previous study has examined these in children growing up in extreme poverty (a specific standard defined by the World Bank as living on less than $1.9 per household member per day) where these risk factors are most severe.

In addition, it remains unclear when functional alterations associated with poverty observed later in childhood (Barch et al., 2016) emerge, and whether they occur during a sensitive period early in life or accumulate over the course of childhood (Jensen et al., 2017; Nelson, 2017). Given that alterations in amygdala iFC in particular have been linked to poor mental health symptoms and outcomes, such as negative emotionality (Graham, Pfeifer, Fisher, Carpenter, & Fair, 2015) and depression (Barch et al., 2016), identifying when and how these alterations emerge could help in preventing or ameliorating these symptoms.

The first year of life represents a period of rapid brain development and heightened neural plasticity (Gao et al., 2009; Gilmore et al., 2012; Knickmeyer et al., 2008; Petanjek, Judaš, Kostović, & Uylings, 2008). It is therefore possible that this period is particularly sensitive to adverse life events  (Fox et al., 2010; Tau & Peterson, 2010), such that the many risk factors associated with poverty are more likely to derail brain development. The only study to quantify poverty‐related functional changes in infants comes from a resting‐state iFC study in 6‐month‐old infants in the U.S., which found that income was positively correlated with iFC between brain areas constituting the functional network and negatively correlated with iFC between constituent brain areas and those outside the functional network for somatomotor and default‐mode networks. However, these effects were not significant after controlling for multiple comparisons (Gao et al., 2015). Furthermore, as this study primarily focused on elucidating changes in canonical networks (e.g. visual, somatomotor, default‐mode networks) over the first year of life, and as the relationship to income was of secondary inquiry, iFC between brain regions previously shown to vary in structure or function depending upon income (e.g. amygdala) was not examined.

**Figure 1:**
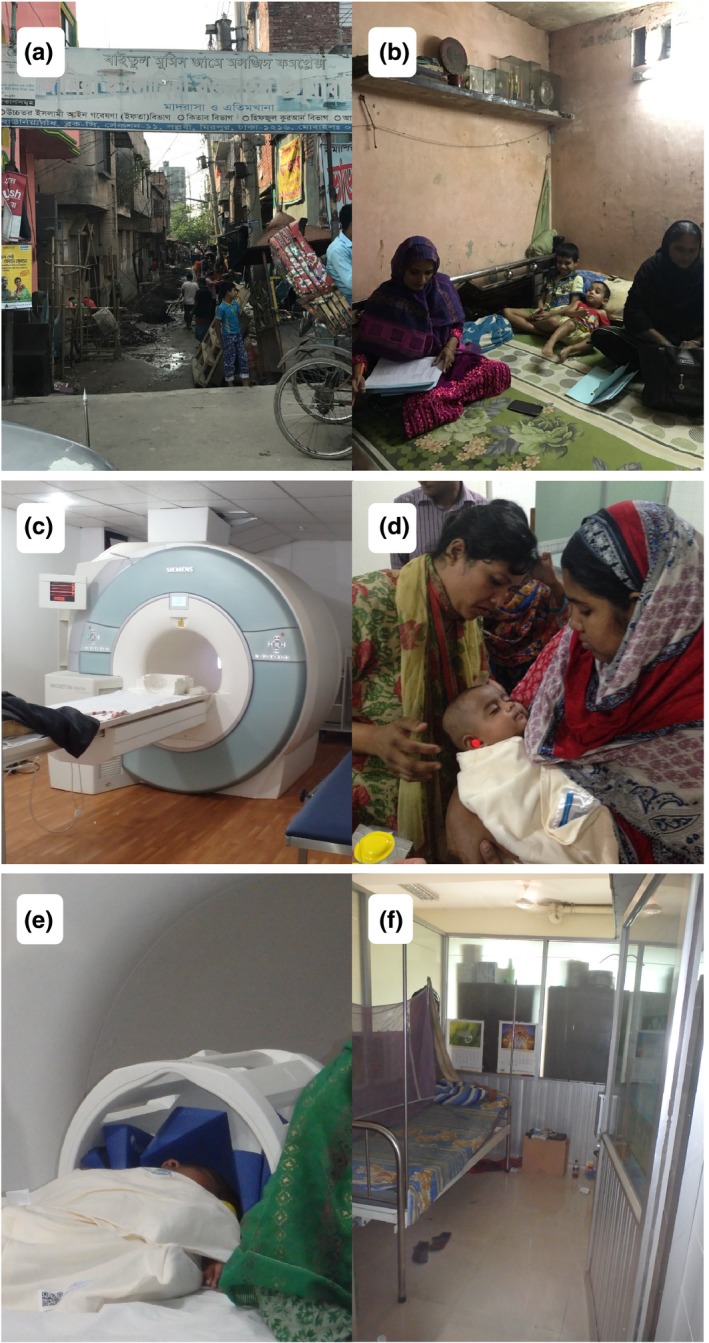
Location of the study. Representative (a) neighborhood and (b) home in Dahka, Bangladesh, where infants in this study reside. (c) MRI facility where infants are scanned. (d) Asleep infant receiving two layers of ear protection. (e) Infant head under head‐coil. As demonstrated, head pads did not tightly secure the infant's head. (f) System administrator's office, used for mothers to put infants to sleep and for final screening

In contrast, the present study focused primarily on amygdala iFC in 2‐month‐old infants growing up in extremely poor environments in Dhaka, Bangladesh (Figure 1a), where severe psychosocial hazards heighten the potential for stress. The amygdala is tied to the stress system through indirect projections to the HPA axis (Gunnar & Quevedo, 2007) and rich expression of receptors for glucocorticoids (Wang et al., 2014), hormones critically involved in stress reactivity. As such, it regulates responses to stress (Doom & Gunnar, 2013), making it a fitting target for examination in this setting. Indeed, one might expect infants living in extreme poverty to exhibit alterations in amygdala function.

The first goal of this study was to examine the feasibility of performing functional MRI in a low‐resource setting. As this was the first infant fMRI study in Bangladesh, an MRI facility needed to be identified; local collaborations needed to be established and sustained; compliance protocols needed to align across all collaborating international institutions; equipment needed to be purchased, shipped, and set up; research staff required intensive training on new equipment, infant imaging techniques, recruitment, and practices for familiarizing infants and their parents with MRI; and data of sufficient quantity and quality needed to be acquired. Scanning was attempted only on a small sample of infants (*n* = 54) because it was not clear whether these steps could be accomplished in Bangladesh. Therefore, this report should be viewed as a feasibility and pilot study.

Previewing later sections of this report, several unique challenges were encountered as part of this study, which were magnified by the special considerations required for infant scanning (Raschle et al., 2012). To aid investigators conducting future studies in low‐resource settings, these challenges and, where possible, solutions are described.

The second goal was to elucidate the potential relationship between biological and psychosocial hazards and early brain development. It is thought that the relationship between family income and brain development is mediated by biological and/or psychosocial hazards, including malnutrition, childhood infections, low maternal education, parental stress, and poor parental mental health (Farah, 2017). For example, low income might restrict a parent's ability to provide nutritious food, which leads to malnourishment and then altered brain development (for a review, please see Jensen et al. (2017)); or, low income may be associated with greater early life (pre‐ and/or postnatal) psychosocial stress (e.g. family conflict), engendering neuroendocrine dysregulation (Arnett et al., 2015) and alterations in brain structure and function (Gianaros & Hackman, 2013; for reviews of the stress response and development, please see Doom & Gunnar, 2013; Gunnar & Quevedo, 2007; Lupien et al., 2009). Identification of specific biological and/or psychosocial factors that contribute to brain alterations is of critical importance so that interventions aiming to reduce the cognitive and emotional consequences of poverty can target those factors.

Achieving this goal involved determining (a) whether there is a relationship between income‐to‐needs and brain function at two months, and (b) whether specific brain areas are particularly vulnerable to certain adverse effects during the first two months of life. Previous reports have linked income‐to‐needs to alterations in amygdala structure and function in older children and adults. Therefore, we hypothesized poverty‐related iFC effects in the amygdala at 2 months‐of‐age. In contrast, sensory brain areas exhibit slow rates of postnatal structural development (Gilmore et al., 2012), and the functional network architecture of these regions at birth is already qualitatively similar to that of adults (Doria et al., 2010; Gao et al., 2015); consequently, we examined sensory iFC with the hypothesis that poverty‐related effects would not be observed.

## 2 METHODS

### 2.1 Participants

The current study is a subset of a much larger study, the Bangladesh Early Adversity Neuroimaging – BEAN – study, investigating the effects of early biological and psychosocial adversity on neurocognitive development among infants and children growing up in Dhaka, Bangladesh (Figure 1a). Whereas the larger study follows 235 infants through infancy into early childhood, the current study focused on a small subset because, as this was the first fMRI study in Bangladesh, obstacles related to scanning in a low‐resource environment (please see subsequent section) needed to be realized before larger cohorts were undertaken.

Unlike with children and adults, infants are scanned while sleeping to avoid motion artifacts without having to use sedation. Replication of typical naptime/bedtime routines in the imaging facility, multiple layers of ear protection, and caregiver proximity during the scan can help to facilitate and maintain infant sleep and mitigate motion during the scan (Raschle et al., 2012). Of 54 infants who attempted MRI, 53 completed the structural/MPRAGE sequence (one infant did not attempt it) and 43 completed the resting‐state/EPI sequence (one infant began the resting‐state sequence but did not finish and 10 did not attempt it). Limited scanner time (2 hr in the morning) presented a challenge, as infants did not always fall (back) asleep during the available time. The scanning success rate in the present study was 80%, which is roughly equal to the 79% rate reported previously for infants <3‐months‐of‐age (Antonov et al., 2017).

Additional participants were excluded based on inadequate image registration (*n* = 2) and excessive inter‐volume motion (*n* = 9), as detailed below. Following these exclusions, 16 infants from families living below the global poverty line (75.1 ± 7.5 days‐of‐age), the criteria for which are detailed below, and 16 infants from relatively higher income families (80.5 ± 10 days‐of‐age) were included in the final analysis (please see Table 1 for demographic details). The proportion of female and male infants differed between extreme poverty and relatively more affluent cohorts (χ^2^(1,*n* = 32) = 6.1; *p *< 0.05), but controlling for sex did not eliminate poverty‐related effects (please see details below). All participants were typical, healthy infants with no diagnosed neurological disease or disability. A clinical radiologist in Bangladesh and a pediatric neuroradiologist at Boston Children's Hospital (BCH) examined structural images to ensure the absence of malignant brain features or abnormalities. The study was approved by research and ethics review committees at BCH and The International Centre for Diarrhoeal Disease Research, Bangladesh (icddr,b).

**Table 1:** Subject demographics

	BPL	APL	p value
*N*	16	16	–
Gender (F/M)	11/5	4/12	<0.05
Age (Days)	75.1 ± 7.5	80.5 ± 10	n.s.
Age Range (Days)	65–90	67–98	–
Income‐to‐Needs^a^	2,700 ± 900	7,500 2,600	<0.0001
Income‐to‐Needs Range^a^	1,300–4000	4,500–13,000	–
HAZ	−1.05 ± 1.1	−1.44 ± 0.97	n.s.
Maternal Education (Years)	3.9 ± 2.9	7.4 ± 3.3	<0.005
Maternal Depression^b^	3.0 ± 4.4	5.8 ± 7.6	n.s.
Maternal Social Isolation^b^	1.7 ± 2.6	1.8 ± 4.9	n.s.
Family Conflict^b^	2.6 ± 3.0	0.92 ± 1.7	n.s.

a Monthly income in Tk (Bangladeshi currency)/ number household members

b Prenatal

### 2.2 Additional considerations for conducting MRI in Dhaka, Bangladesh

The present study involved a collaboration among multiple facilities (BCH, University of Virginia, icddr,b, the National Institute of Neurosciences & Hospital – NINSH) and many personnel at these facilities. The dependence on multiple international facilities complicated operations for several reasons. First, language differences between teams at BCH and in Bangladesh presented challenges for technical training and troubleshooting MRI sequences. Second, differences between Boston and Dhaka in time zone (11 hr) and days designated as weekends (Saturday and Sunday vs Friday and Saturday) reduced weekday overlap between BCH and Bangladesh facilities to three days. Third, institutional compliance practices needed to be standardized across participating institutions. For example, although institutions in many countries, including the U.S., sedate infants for clinical scans, sedation for research purposes is considered unethical in these countries. NINSH initially questioned this regulation because they did not have experience scanning infants for research purposes. Fourth, political tensions at times limited the investigators from the U.S. who could travel to Bangladesh to collaborate in‐person. Finally, transfer of MRI data became a multistep process because the scanner is not connected to the internet.

Recruitment was conducted differently from studies in higher‐resource countries. Herein, a neighborhood (in this case the Mirpur district of Dhaka) was selected by project directors and census data were gathered to determine which mothers were pregnant. Staff from icddr,b were trained to describe to families the eligibility requirements and procedures for participating in an MRI study. Once trained, they contacted potential participant families by visiting their homes, rather than by distributing flyers, calling, or emailing. Study‐specific field sites affiliated with icddr,b, which were established in the Mirpur district for the overarching BEAN project, were used to consent and conduct behavioral testing on participating mothers and infants. Language differences necessitated that all eligibility and consent forms, study descriptions, and behavioral batteries be translated from English to Bengali.

The MRI facility at NINSH (Figure 1c) also required preparation for collecting infant data. The scanner itself is an older model than the scanners used at most medical centers in the U.S. To mitigate this difference, a research assistant was scanned in Boston and Dhaka and sequences were fine‐tuned for the NINSH scanner. Infants were prepared for the scan using locally purchased in‐ and over‐ear noise protection (Figure 1d), Halo SleepSacks^®^ for swaddling, cut and fitted extra‐thick memory foam to pad the scanning bed, and multiple MRI‐safe head‐pads to secure infants’ heads inside an adult‐sized head‐coil (please see Figure 1e for visualization of this problem); washcloths were added when head‐pads were insufficient.

Staff at NINSH had prior experience scanning infants for clinical, but not for research, purposes. For additional training, they traveled to Boston to observe pediatric neuroimaging sessions and practice positioning pediatric participants in the MR scanner. Importantly, techniques used to ensure infants sleep (and remain still) during scans are multiplex (Antonov et al., 2017; Dean et al., 2014; Hughes et al., 2017). The execution of these in Bangladesh was complicated by two major factors: one was that naptimes are unstructured in Bangladesh, and two, travel from homes to NINSH is difficult because families do not own cars. To address this, staff in Dhaka and at BCH collaboratively generated a location‐specific plan to prepare families for scans in such a way as to maximize the likelihood that infants would sleep during the scan. A key component of this plan was that staff would escort families to NINSH in taxis and promote wakefulness during transit. Severe traffic in Dhaka, which prolonged transit, intensified this challenge.

Availability of time and space at NINSH was also problematic. The MRI scanner was only available for research purposes 7:00 a.m.–9:00 a.m., two days per week. Also, NINSH lacked a dedicated room for mothers to put their infants to sleep. The system administrator's office was appropriated for this purpose, though it was not equipped to play MRI sounds at low volume and could not be dimly lit (Figure 1f), two techniques used by BCH staff to induce sleep in infants in the U.S. (Raschle et al., 2012).

Lastly, staff in this low‐resource setting seemed to have more involvement with families than staff in high‐resource settings. In‐person recruitment, escorting to and from NINSH, and clear descriptions of expectations for MRI studies are examples of this. Indeed, the close ties of the research staff to the community were critical to the feasibility of this study.

### 2.3 Measures of poverty and commonly associated biological and psychosocial risk factors

The World Bank (https://data.worldbank.org/) has set the international extreme poverty standard at income‐to‐needs (i.e. the daily household income divided by the number of household members) at USD$1.90 per person per day. Infants were initially recruited from two neighborhoods in Dhaka, which were chosen to broadly reflect a difference in socioeconomic class. However, some infants from the “lower class” group were from families earning above the World Bank threshold and some infants from the “middle class” group were from families earning below this threshold. To specifically examine poverty‐related brain effects and identify mediating effects by risk factors associated with poverty, infant cohorts were restructured roughly according to the World Bank standard for extreme poverty. To maintain equal group sizes, a threshold slightly lower than that used by the World Bank was employed: USD$1.80. With an exchange rate of USD$1:Tk80 (Bangladeshi taka), this meant that infants from families earning less than or equal to Tk4320/USD$54 per month per household member were designated “below the poverty line” (BPL) infants, and infants from families earning greater than Tk4320 per month per household member were designated as “above the poverty line” (APL) infants. Maternal education was measured as years of formal education, ranging from 0 to 10 (0 indicated no formal education, 1‐9 indicated number of classes passed, and 10 indicated education beyond the 9th class passed).

Household income, number of household members, and maternal education were reported at the time of enrollment in the study (i.e. shortly after birth or at 2 months‐of‐age) through oral interviews with the mothers of the infants. In a subsample of infants (*n* = 22), mothers were administered a questionnaire capturing the prenatal psychosocial environment; scores were acquired for maternal depression, maternal social isolation, and family conflict (including emotional abuse). These scales were pulled from a new tool to assess psychosocial adversity in global low‐resource settings that was under development as part of the overarching study. Questions were adapted from previously validated scales (e.g. the Edinburg Postnatal Depression Scale) and altered according to input from focus groups constituting professionals and mothers involved in the broader BEAN study. The benefit of this adapted questionnaire over preexisting questionnaires is that it is culturally appropriate for the low‐resource setting of Bangladesh (Berens et al., n.d.). The reason 10/32 infants did not complete this questionnaire is because it was not finalized until after these 10 infants had returned for their 6‐month visit, when the questionnaire was designed to be administered. Trained, local staff conducted interviews and examinations. Infant height relative to an age‐ and sex‐specific growth reference (i.e. height‐for‐age; HAZ) was used as an indicator of diminished growth that may reflect malnutrition and/or exposure to infectious disease (de Onis & Branca, 2016; de Onis, Garza, Victora, Bhan, & Norum, 2004). Height was measured in centimeters at the time of the scan and then converted into a standardized HAZ score using the World Health Organization's Anthro Plus software (version 3.2.2). Table 1 summarizes these measures in our cohorts.

### 2.4 MRI data acquisition

All neuroimaging data were acquired while infants were asleep in a 3T Siemens MAGNETOM Verio scanner at NINSH in Dhaka, Bangladesh (Figure 1c). Structural T_1_‐weighted magnetization‐prepared rapid gradient‐echo MPRAGE scans were acquired with the following parameters: TR = 2,520 ms, TE = 2.22 ms, 144 sagittal slices, 1 mm^3^ voxels, FOV = 192 mm. Gradient‐echo echo planar imaging (EPI) was used to acquire 205 functional, blood‐oxygen‐level dependent (BOLD) volumes. The initial resting‐state sequence used the following parameters: TR = 2,310 ms, TE = 30 ms, 36 3 mm‐thick axial slices with no gap acquired interleaved, in‐plane resolution of 64 × 64 (3 mm isotropic voxels), and FOV = 192 mm. However, when technicians realized that this sequence was not capturing the whole brain in a subset of infants, the number of slices was increased from 36 to 44 and the TR was elongated to 2,820 ms from 2,310 ms to preserve the same number of volumes per subject (205); total scan times were 7 min 54 s and 9 min 38 s respectively. In the final cohort, only four infants exhibited an insufficient number of slices to cover the dorsal‐most aspect of the brain (mainly in medial somatomotor cortex), with 3/4 missing 4 mm or less and 1/4 missing roughly 12 mm when subtracting the dorsal‐most aspect of the MPRAGE image with the dorsal‐most aspect of the EPI image.

We undertook additional steps to ensure that our fMRI result (previewing the Results section – iFC between amygdala and left precuneus) was not confounded by an insufficient number of slices in these four subjects. First, warping to standard space was done using T1 images, which did not exhibit any loss of brain from the FOV; consequently, when this transform was applied to the EPI images, placement of brain regions was maintained without stretching. And second, pre‐processed images for the four participants with an insufficient number of slices were overlain with the between‐group map. For all four infants, brain tissue in the preprocessed images overlapped with the left precuneus cluster revealed in the between‐group comparison.

We also undertook steps to ensure that the fMRI result was not confounded by differences in resting‐state sequence in general, regardless of whether there were insufficient numbers of slices in some subjects who underwent the first sequence. First, between‐group analyses were recomputed with groups designated by sequence rather than by extreme poverty. iFC differences were not observed in any brain regions at *p *= 0.01 height threshold, *p *= 0.1 *FDR* cluster‐level correction. Second, second‐level analyses were recomputed with the independent variable modeled continuously with income‐to‐needs. A similar precuneus cluster was observed at the same *FDR* cluster‐level correction of *p *< 0.05, but slightly more lenient height threshold of *p *< 0.005. And third, to control for differing parameters, partial correlations were used to ensure that the relationship between income‐to‐needs and any resulting brain‐based effects remained significant after controlling for scanner parameters (please see below for details). Nevertheless, combining resting‐state datasets acquired with differing parameters, and including a small subset of EPI images that do not cover the whole brain in some subjects, would be atypical and concerning if collected in a high‐resource setting. Consequently, results should be interpreted with caution.

### 2.5 MRI data analyses

#### 2.5.1 MPRAGE preprocessing

Raw structural images were first bias‐corrected using N4ITK (Tustison et al., 2010) implemented in Advanced Normalization Tools (ANTS). To account for inter‐subject brain structural differences, images were then warped to the neonate template (with cerebellum) from the Biomedical Research Imaging Center (BRIC) at the University of North Carolina (UNC; Shi et al., 2011) using the antsRegistrationSyNQuick module (Avants et al., 2011), consisting of rigid, affine, and deformable b‐spline Symmetric Normalization (“SyN”) transformations. This procedure, consistent with an earlier resting‐state fMRI study in infants (Merhar et al., 2016), produced transforms for each participant, which were later applied to each participant's EPI images, as described below.

In parallel, raw structural images were submitted to a dedicated infant version of the FreeSurfer recon‐all pipeline (Zöllei, Ou, Iglesias, Grant, & Fischl, 2017) for segmentation into 44 cortical and subcortical gray and white matter regions and ventricles, all in native space. This pipeline (https://surfer.nmr.mgh.harvard.edu/fswiki/infantFS) takes a multi‐atlas approach and selects relevant atlas information to use depending on its similarity to the brain to be segmented. A prior‐based Bayesian approach (Iglesias, Sabuncu, & Leemput, 2012) was used with a training dataset of infant structural images in which brain areas, such as the amygdala, were individually and manually segmented (de Macedo et al., 2015). Deformable Registration via Attribute Matching and Mutual‐Saliency Weighting (DRAMMS) (Ou, Sotiras, Paragios, & Davatzikos, 2011) was used as part of registering atlas and subject data. It should also be noted, however, that there is still no consensus about the segmentation pipeline for infant imaging (Makropoulos, Counsell, & Rueckert, 2018; Zhang, Shen, & Lin, 2019). These segmentations were subsequently consolidated into gray matter (GM), white matter (WM), and cerebrospinal fluid (CSF) regions‐of‐interest (ROIs) for each participant using in‐house MATLAB 2015b (*MathWorks*) code. The transforms derived in the previous step were combined and applied to the segmentations, warping them from native to standard space in one interpolation using the antsApplyTransforms module. Warped subject images were visually inspected alongside the UNC template to ensure alignment.

#### 2.5.2 EPI Preprocessing

To reduce T1 saturation effects, the first 10 volumes from each resting‐state run were discarded, as in Gao and colleagues (2015), reducing the number of volumes per subject from 205 to 195. Using SPM12 (http://www.fil.ion.ucl.ac.uk/spm/), volumes were (a) slice‐time corrected to account for sampling superior and inferior parts of the brain at different times and (b) realigned to correct for inter‐volume head motion throughout the run, which also output six rigid body parameters. Again using ANTS, EPI images were subsequently coregistered to T1 images and normalized in one interpolation using the transforms generated in warping participant MPRAGE images to the UNC neonatal template (Shi et al., 2011). As in prior infant fMRI studies (Fransson et al., 2007; Merhar et al., 2016), warped EPI images were smoothed in SPM using a 6.0 mm full width at half maximum Gaussian kernel to improve the signal‐to‐noise ratio.

Three additional quality control steps were undertaken to account for in‐scanner head motion, since iFC estimates are particularly susceptible to head motion artifacts (Van Dijk, Sabuncu, & Buckner, 2012; Satterthwaite et al., 2013), especially in younger ages (Satterthwaite et al., 2012). First, we removed participants for whom ≥20% of the volumes from the resting‐state run were preceded by inter‐volume head motion ≥0.5 mm (17% of the voxel size) root‐mean‐square (RMS) displacement (i.e. d^2^=∆x^2^+∆y^2^+∆z^2^+[(30π/180)^2^·(∆pitch^2^+∆roll^2^+∆yaw^2^)], similar to other infant studies (Gao et al., 2015). RMS displacement has been used previously in the context of functional connectivity (Van Dijk et al., 2012) and was implemented using the ArtRepair tool (Mazaika, Whitfield, & Cooper, 2005). Calculating RMS requires converting from degrees to millimeters, which requires an estimation of brain radius. Power and colleagues (2012) measured brain radius as “approximately the mean distance from the cerebral cortex to the center of the head.” Consistent with this procedure, an estimation of 30 mm was empirically measured by subtracting the z‐coordinates corresponding to the center of the brain and cerebral cortex of the UNC template; this distance was then employed in the RMS calculation. This procedure removed nine infants from the sample, which, along with the two infants removed due to poor registration, resulted in a total of 32 infants for the final analyses. Second, volumes that were preceded by ≥0.5 mm inter‐volume head motion were entered as temporal confounding factors (please see below) to ensure that any variance associated with these volumes was effectively removed. And third, BPL and APL cohorts were compared on seven inter‐volume head motion variables: mean and maximum (a) translation, (b) rotation, and (c) overall (i.e. translation and rotation combined) RMS displacement (after removing runs in which ≥20% of volumes were preceded by excessive head motion and, in the remaining runs, volumes preceded by ≥0.5 mm head motion), and (d) number of volumes removed from participants in the final sample (Table 2). Two‐sample *t* tests revealed no significant differences between BPL and APL cohorts (*p *> 0.05).

**Table 2:** In‐Scanner head motion

	BPL	APL
Mean Inter‐Volume Translation (mm)	6.4 (2.1)	7.2 (2.0)
Max Inter‐Volume Translation (mm)	36 (9.3)	36 (9.8)
Mean Inter‐Volume Rotation (mm)	3.0 (1.0)	3.7 (1.2)
Max Inter‐Volume Rotation (mm)	25 (10)	25 (11)
Mean Inter‐Volume Overall (mm)	7.3 (2.3)	8.3 (2.2)
Max Inter‐Volume Overall (mm)	40 (9.9)	40 (9.3)
Number of Volumes Removed	8.19 (11)	11.5 (9.9)

Note

Measures are mean and standard deviations. All values × 10^−2^. Measures were calculated after discarding volumes preceded by ≥0.5 mm inter‐volume head motion and after discarding runs in which 20% or more of the volumes were preceded by ≥0.5 mm inter‐volume head motion.

All subsequent image processing was undertaken using CONN 17f (https://www.conn-toolbox.org/; Whitfield‐Gabrieli and Nieto‐castanon (2012). To maximize signal‐to‐noise, all warped, smoothed functional data (following preprocessing and motion quality control steps detailed earlier) were denoised, which involved regression of temporal confounding factors followed by temporal filtering (this ordering is explained in Graham et al., 2015; Graham et al., 2016). Temporal confounding factors included six rigid body head position parameters output from the realignment step of preprocessing, a logical matrix to indicate whether scans were preceded by ≥0.5 mm (i.e. scans preceded by inter‐volume head motion <0.5 mm received a 0 and scans preceded by inter‐volume head motion ≥0.5 mm received a 1), and the effect of the rest condition convolved with the canonical hemodynamic response function, in case ramping effects persisted beyond the 10 volumes discarded at the beginning of the run. Importantly, the second temporal confounding factor above is consistent with the motion artifact “scrubbing” procedure described by Power and colleagues (2012) and generated using ArtRepair. To further remove physiological noise associated with the BOLD signal, but without introducing spurious negative correlations resulting from global signal regression (Chai, Castañán, Öngür, & Whitfield‐Gabrieli, 2012), CONN implements the CompCor method (Behzadi, Restom, Liau, & Liu, 2007). Herein, principal components are estimated using all voxels contained within participant‐specific WM and CSF ROIs, which were created in the segmentation step (described in the MPRAGE preprocessing section). Five principal components per participant designated for WM and CSF were entered as additional temporal confounding factors. A band‐pass filter of 0.008–0.09 Hz was used, which is consistent with another infant resting‐state study examining adversity (Graham et al., 2015). A range similar to this one has been encouraged to reduce iFC estimates related to motion (Satterthwaite et al., 2013). This produced denoised BOLD time series for every voxel of each participant dataset.

#### 2.5.3 Resting‐state functional connectivity

All functional connectivity analyses were performed using a seed‐to‐voxel approach. Similar to a previous study (Merhar et al., 2016), seeds were localized using the Neonate Automated Anatomical Labeling (AAL) atlas, which is the AAL map originally defined in Montreal Neurological Institute (MNI) stereotaxic space warped longitudinally via 2‐year‐old brains to the neonate brain space. Developed by BRIC at UNC, this atlas was registered to the structural template used in the normalization step described earlier. Given the convergence of previous literature on poverty‐related alterations in the amygdala and ability of sensory iFC to inform on spatial and temporal specificity of poverty‐related effects (please see Introduction), bilateral seeds were placed in the amygdala, calcarine cortex, and Heschl's gyrus (6 seeds total). Next, denoised BOLD time series for voxels constituting these seeds underwent single value decomposition (SVD), yielding one (eigenvariate) time series for each seed. In the adult brain, signal heterogeneity exists within brain regions labeled according to the AAL atlas (Gordon et al., 2016). This can be problematic when attempting to reduce voxel time series within a region to one time series: e.g. averaging would produce a seed time series that is different than any of the constituent voxels’ signals. In contrast, SVD addresses this heterogeneity by separating constituent signals into eigenvariates, with the first eigenvariate representing the dominant signal within the seed. Participant‐level whole‐brain seed‐to‐voxel maps were then generated, in which each voxel represents the bivariate correlation (reported as Fisher's z‐scores) of that voxel's denoised time series with the time series of the seed. Within‐group brain maps were generated for bilateral seed pairs (e.g. left and right Heschl's gyri) using a repeated measures design, in which iFC estimates for homologous seed regions were combined into one model. Generation of between‐group brain maps similarly relied on a main effect of bilateral seeds combined, but were modeled to identify effects of poverty (BPL>APL and AP>BPL contrasts). Resultant clusters from these second‐level analyses were reported significant using an *FDR* cluster‐level correction of *p *< 0.05 and an uncorrected height threshold of *p *< 0.001.

Next, participant‐level average (across voxel) iFC estimates extracted using the REX toolkit (http://web.mit.edu/swg/software.htm) from clusters significant in between‐group analyses were submitted to additional correlation analyses. To ensure that effects in these clusters were related to income‐to‐needs and not another variable shown to differ between groups (please see Table 1), partial correlations were run on income‐to‐needs (used to define groups and designate participants as BPL or APL, but treated here as a continuous variable) and extracted iFC estimates while controlling for differing ratios of (a) two different resting‐state sequences (please, see MRI acquisition section) and (b) female/male participants. These ROI‐level partial correlations remained significant (*p *< 0.05), suggesting that the between‐group result was not confounded by these other variables.

Lastly, additional analyses were conducted to determine whether the biological and psychosocial risk factors described earlier could explain the relationship between income‐to‐needs (treated in these analyses as a continuous variable) and participant‐level iFC estimates extracted from the between‐group comparison. First, the sample size was reduced to *n* = 22 (i.e. imputation was not used) because the psychosocial environment questionnaire for prenatal maternal depression, maternal isolation, and family conflict was not finalized by the time 10/32 infants reached 6‐months‐of‐age, which is when the questionnaire was administered. Second, D'Agostino & Pearson omnibus normality tests performed on income‐to‐needs and iFC estimates showed that these data were not distributed differently from normal (income‐to‐needs: K2 = 1.66, *p *> 0.05; amygdala/precuneus iFC: K2 = 1.39, *p *> 0.05), justifying the use of parametric testing for brain‐risk factor analyses. Third, bivariate cross‐correlations were performed between the primary independent variable (income‐to‐needs), risk factors (HAZ, maternal education, and prenatal psychosocial scores), and the dependent variable (iFC), using JASP software (https://jasp-stats.org/). For a variable to be mediating, it must be significantly correlated with both independent and dependent variables. However, none of the risk factors fit this requirement. Finally, these variables were submitted to a stepwise multiple regression analysis to determine which of the risk factors contributed unique variance. Semi‐partial correlation values were squared to compute percentage of unique variance.

#### 2.5.4 Reporting and visualization

To determine the location of significant clusters, second‐level brain maps were overlaid on the UNC Neonate AAL atlas (Shi et al., 2011). For the between‐group brain map, a quantitative approach was used to determine the percentage of voxels falling into specific brain areas. All within‐group brain maps were visualized using the CONN graphics interface and between‐group maps were visualized using the Mango software package (http://rii.uthscsa.edu/mango/) with the UNC neonate template (Shi et al., 2011). All graphs were visualized using GraphPad Prism 6.

## 3 RESULTS

### 3.1 Within‐group iFC maps

**Figure 2:**
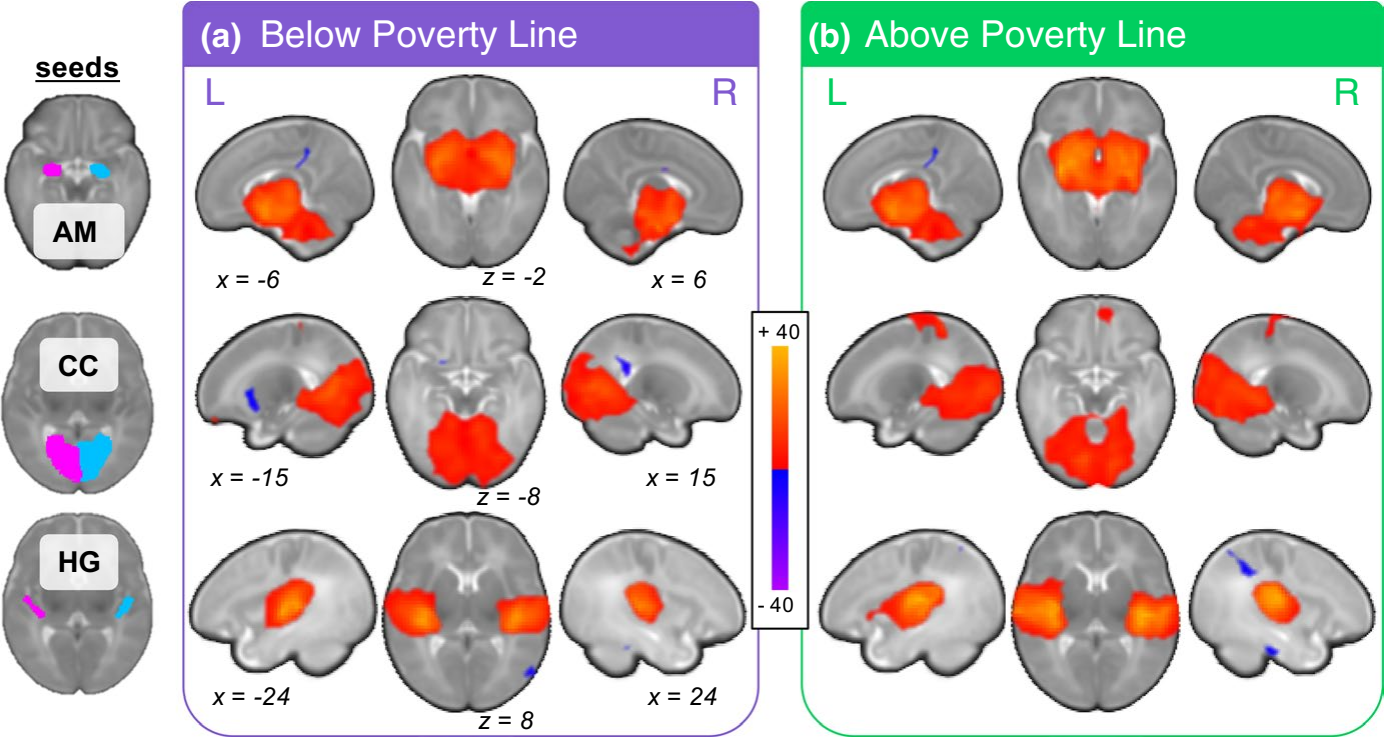
Within‐group iFC maps for infants (a) below and (b) above the poverty line. Neonatal atlas specifications for amygdala (row 1), calcarine cortex (row 2), and Heschl's gyrus (row 3) served as seed regions. Coordinates of slices shown in the BPL box also apply to the APL group maps. All maps were thresholded with *FDR* cluster‐level correction of *p *< 0.05. Colorbar reflects positive (red/yellow) and negative (blue) iFC (t‐statistic). L, left hemisphere; R, right hemisphere; AM, amygdala; CC, calcarine cortex; HG, Heschl's gyrus. Table 3 provides the full list of brain areas with iFC to these seeds

Whole‐brain iFC was examined using bilateral seeds in the amygdala, calcarine cortex, and Heschl's gyrus (3 seed pairs). As such, within‐group maps illustrate brain areas whose time series correlate positively (Figure 2, red/orange) and negatively (Figure 2, blue) with the time series of the seed pair (e.g. bilateral Heschl's gyrus). Because iFC estimates for most seed pairings were not significantly different between groups, within‐group results for BPL and APL cohorts are reported below together. For a full list of brain areas constituting these iFC maps, please see Table 3.

**Table 3:** Within‐Group iFC peaks

Group	Anatomical region	Peak MNI coordinate			k	Z
*Seed*		x	y	z		
BPL						
*Amygdala Seeds*	L. Amygdala	−18	−4	−8	7,154	Inf
	L. Precuneus	−10	−46	38	80	3.75
	R. Middle Cingulate Gyrus^a^	6	−4	20	106	−4.06
*Calcarine Cortex Seeds*	L. Calcarine Cortex	−4	−48	4	8,663	6.85
	R. Middle Frontal Gyrus^a^	28	−2	42	97	4.8
	L. Postcentral Gyrus	−24	−20	46	167	4.73
	L. Orbitofrontal Cortex Superior	−8	34	−16	95	4.56
	L. Insula	−16	4	−6	120	−4.22
	R. Posterior Cingulate Gyrus^a^	14	−28	16	167	−4.9
*Heschl's Gyrus Seeds*	L. Insula	−24	−12	4	1851	7.57
	R. Heschl's Gyrus	28	−16	8	1594	7.32
	R. Precuneus	12	−32	26	83	−4.25
	R. Middle Temporal Gyrus	36	−46	4	295	−4.29
APL						
*Amygdala Seeds*	L. Insula	−16	6	−6	8,659	Inf
	L. Supramarginal Gyrus	−32	−26	22	96	−3.76
	L. Middle Cingulate Gyrus	0	−20	18	161	−4.09
	R. Superior Temporal Gyrus^a^	48	−20	8	83	−4.2
	L. Inferior Temporal Gyrus	−32	−10	−18	77	−4.86
*Calcarine Cortex Seeds*	R. Lingual Gyrus	4	−50	−2	8,973	6.46
	L. Superior Parietal Gyrus	−16	−30	44	1561	4.85
	L. Cerebellum	−4	−38	−18	63	4.13
	L. Superior Frontal Gyrus	−8	40	16	143	3.96
	R. Orbitofrontal Cortex Superior	10	34	−6	89	3.78
	L. Rectus Gyrus^a^	−2	24	−22	68	3.7
	R. Postcentral Gyrus^a^	20	−28	20	69	−3.96
	R. Insula	24	−2	12	217	−4.34
	L. Angular Gyrus^a^	−36	−52	22	81	−4.44
	L. Inferior Frontal Gyrus Opercular^a^	−22	−2	16	114	−4.57
*Heschl's Gyrus Seeds*	R. Rolandic Operculum	26	−16	12	2,871	Inf
	L. Heschl's Gyrus	−26	−16	10	2,577	Inf
	R. Inferior Temporal Gyrus	26	−16	−18	105	−4.05

a Nearest gray matter according to neonate atlas.

For amygdala seeds, iFC was observed with one large, bilateral cluster comprising hippocampus, parahippocampal gyrus, insula, basal ganglia, and thalamus, consistent with a previous report in 6‐month‐old infants (Qiu et al., 2015).

Sensory seeds were subsequently investigated. For calcarine cortex seeds, positive iFC was observed with other areas of occipital cortex and anterior frontal cortex; negative iFC was observed between calcarine seeds and bilateral angular gyrus and left insula (peak in left putamen). Heschl's gyrus seeds exhibited positive iFC with bilateral superior temporal gyrus and negative iFC with right inferior/middle temporal gyrus and right inferior parietal lobe (for the APL group, as an extension of a right hemisphere temporal lobe cluster).

### 3.2 Between‐group IFC map

#### 3.2.1 BPL>APL

**Figure 3:**
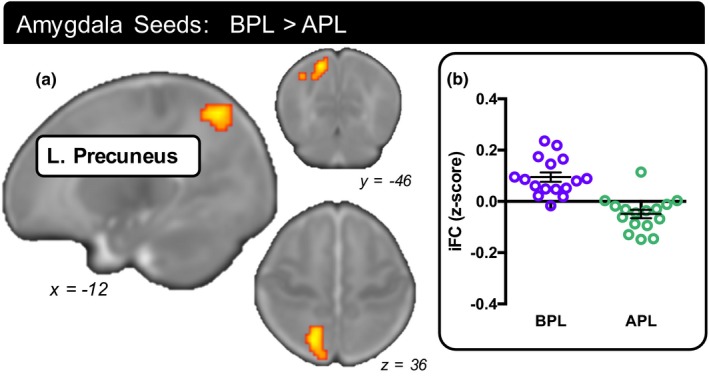
Whole‐brain iFC from BPL>APL contrast. (a) This contrast identified only one brain region in which BPL infants exhibited greater iFC with bilateral amygdala seeds compared with APL infants: left precuneus (k = 177 voxels); *p *< 0.05 *FDR* cluster‐level corrected. (b) Individual iFC estimates extracted from the left precuneus cluster are depicted to the right of the brain map for differentiating positive and negative within‐group iFC and showing inter‐participant variance. L, left hemisphere; R, right hemisphere

BPL compared with APL infants exhibited greater iFC between amygdala seeds and left precuneus (60% of k = 177 voxels; peak coordinates in left superior parietal cortex: x = −12, y = −46, z = 36), extending into left inferior and superior parietal lobules (24%) and left middle and superior occipital gyri (16%; Figure 3a). Individual iFC measures extracted from left precuneus showed that this cluster positively correlates with the amygdala seeds in most BPL infants (qualitatively) and in the BPL group on average (t(15)=5.19; *p *< 0.0005), while the APL group on average (t(15) = 2.99; *p *< 0.01) and all except for one APL infant (qualitatively) exhibited negative iFC in this region (Figure 3b). iFC did not differ by group for any other homologue seed pairings.

#### 3.2.2 APL>BPL

No brain areas exhibited greater iFC with any of the homologue seed pairings in the APL compared with BPL contrast.

### 3.3 Association of biological and psychosocial risk factors of poverty to amygdala/precuneus iFC estimates

**Figure 4:**
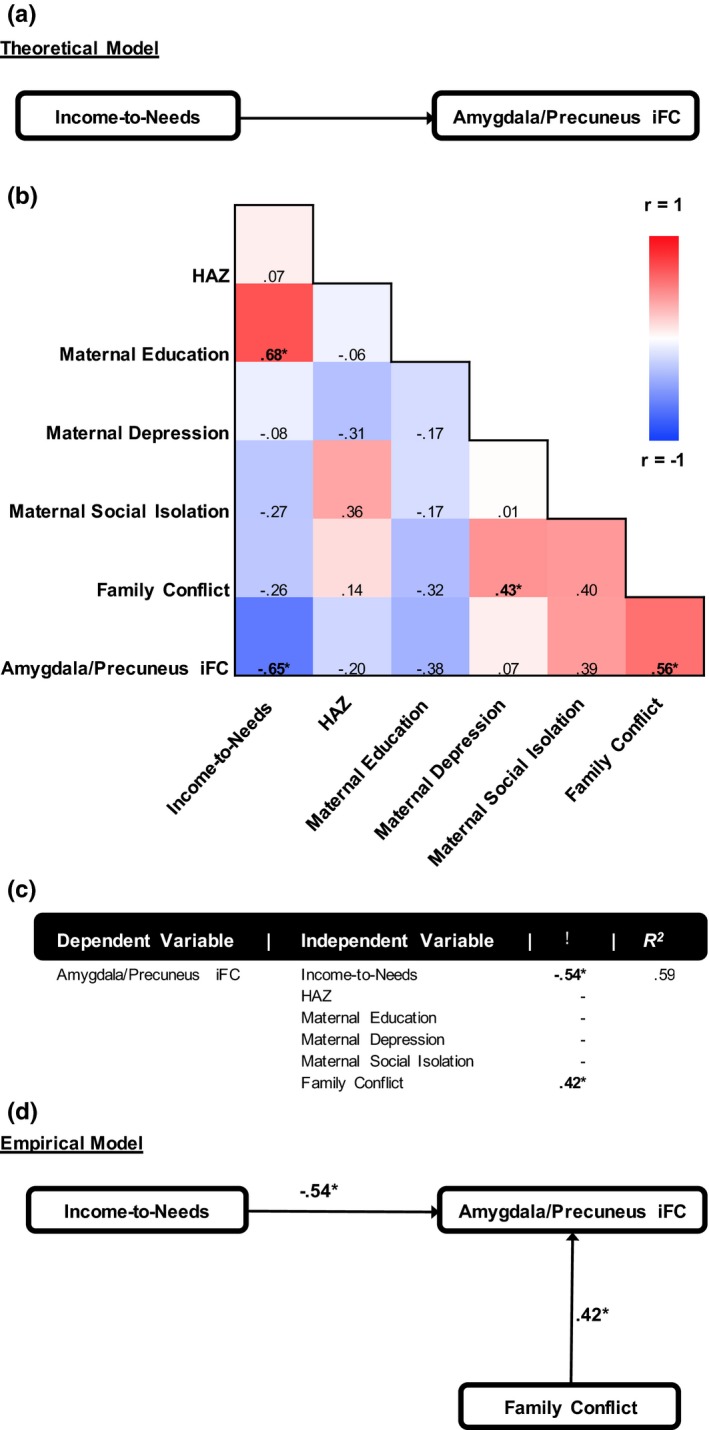
Steps toward modeling mediation using biological and psychosocial risk factors. (a) A preliminary theoretical model based on the between‐group result was proposed relating income‐to‐needs and amygdala/precuneus iFC. (b) Correlation matrix between income‐to‐needs, risk factors, and amygdala/precuneus iFC shows positive (red) and negative correlations (blue). Because prenatal psychosocial variables were collected for only 22 infants, all other variables for the remainder of this section were also reduced to 22. (c) Results of the stepwise multiple regression in which only income‐to‐needs and prenatal family conflict uniquely explained variance in amygdala/precuneus iFC estimates. (d) Preliminary empirical, data‐driven model depicting regressions between income‐to‐needs and amygdala/precuneus iFC as well as between family conflict and amygdala/precuneus iFC. All beta values are standardized. Bold font and * indicates *p *< 0.05

From the between‐group result, a preliminary theoretical model of the relationship between income‐to‐needs and amygdala/precuneus iFC alterations was proposed (Figure 4a). A data‐driven approach to building a model was subsequently undertaken to explore whether risk factors commonly associated with poverty in low‐resource settings like urban Bangladesh are also associated with amygdala/precuneus iFC. Namely, HAZ was used as a proxy for malnutrition and/or exposure to infectious disease; low maternal education, prenatal maternal depression, prenatal maternal isolation, and prenatal family conflict were employed as psychosocial measures. Importantly, these analyses relied on 22 datasets (please see Methods) and should be considered preliminary given the constraints imposed by a small sample size (Poldrack et al., 2017). A correlation matrix was generated to determine which of these risk factors correlated with both income‐to‐needs (here treated as a continuous variable) and amygdala/precuneus iFC taken from the between‐group result above (Figure 4b). Maternal education was correlated with income‐to‐needs and prenatal family conflict was correlated with amygdala/precuneus iFC. A variable must be correlated with both independent and dependent variables to be considered mediating. But as shown, no risk factor correlated with both income‐to‐needs and amygdala/precuneus iFC, precluding subsequent mediation testing.

To determine whether prenatal family conflict explained amygdala/precuneus iFC variance unique from income‐to‐needs, risk factors were also submitted to a stepwise multiple linear regression with amygdala/precuneus iFC as the dependent variable. This set of independent variables explained 59% of the variance (*F*(2,19) = 14; *p *< 0.001) in amygdala/precuneus iFC. Importantly, after controlling for each other and other risk factors, income‐to‐needs and family conflict uniquely explained 27% (standardized β = −0.54, *p *< 0.005) and 16% (standardized β = 0.42, *p *< 0.05) of iFC variance, respectively (Figure 4c); this, along with no significant correlation between income‐to‐needs and family conflict, supported modeling these variables as separately associated with amygdala/precuneus iFC (Figure 4d).

## 4 DISCUSSION

The present study is the first infant MRI study undertaken in the low‐resource setting of Bangladesh. A small, pilot sample of infants was recruited to determine whether an fMRI study of this kind is feasible in a low‐income setting. Feasibility depended upon identifying a reliable scanning facility; establishing and sustaining local collaborations; aligning all collaborating international institutions’ compliance practices; setting up study‐specific equipment; training research staff on new equipment, infant imaging techniques, recruitment, and practices for familiarizing infants and their parents with MRI; and collecting data of sufficient quantity and quality. These steps were accomplished, suggesting that it is feasible to conduct infant MRI studies in low‐resource settings such as Bangladesh. Providing more detailed training for technicians who are unfamiliar with MRI studies could improve data quality for future studies in low‐resource settings.

This is also the first MRI study to examine iFC in infants experiencing profound poverty and associated biological and psychosocial hazards. Bilateral seeds in the amygdala, calcarine cortex, and Heschl's gyrus were used to generate medial temporal/striatal (Qiu et al., 2015) and canonical visual and auditory resting‐state networks, as shown in previous studies in infants (Doria et al., 2010; Fransson, Åden, Blennow, & Lagercrantz, 2011; Fransson et al., 2007; Gao et al., 2015; Gao, Lin, Grewen, & Gilmore, 2017) and adults (e.g. Cole, Smith, & Beckmann, 2010; Damoiseaux et al., 2006) from high‐resource countries. When directly compared with infants above the global poverty line (APL), infants from extreme poverty (BPL) exhibited greater (i.e. less negative) iFC between bilateral amygdala seeds and left precuneus. The negative participant‐level iFC estimates for the APL group, as extracted from the between‐group result, align with previous findings showing that negative amygdala/precuneus iFC is typical in adults (Zhang & Li, 2012) and that *attenuated* negative amygdala/precuneus iFC is associated with *greater* symptoms of depression in children (Barch et al., 2016). Amygdala/precuneus iFC was not associated with HAZ, a proxy for malnutrition and exposure to infection (de Onis & Branca, 2016; de Onis et al., 2004). However, prenatal family conflict accounted for variance in amygdala/precuneus iFC that was unique from variance explained by income‐to‐needs, suggesting a relationship between this psychosocial risk factor commonly associated with poverty and altered brain function in the amygdala. Lastly, these results suggest that the relationship between income‐to‐needs and brain function is detectable even by the first 2 months following birth within specific functional connections, one of which is between amygdala and precuneus.

### 4.1 Expectations for conducting infant fMRI in Dhaka, Bangladesh

There were several challenges associated with the present study that should be mentioned to facilitate developmental cognitive neuroscience studies in low‐resource countries. First, institutions in Bangladesh lacked resources essential for undertaking infant fMRI studies, particularly equipment and trained staff. Similarly, the MRI scanning facility had limited availability. Second, recruitment needed to be conducted in person, by visiting homes of potential study participants, as electronic communication was not always available. However, this can also be considered a strength of the present study as in‐person communication led to better relationships with families, which in turn diminished hindrances with recruitment and retention. Indeed, all infants who participated in the present fMRI study at 2‐months‐of‐age returned at 6‐months‐of‐age for other study components. Third, implementing this study required the coordination of investigators in Boston and research staff at two sites in Dhaka, which was challenging due to differences in time zone, weekend and holiday schedules, and languages. Fourth, maintaining data quality standards as established for high‐resource countries was challenging. For instance, in the present study, staff changed resting‐state sequence parameters partway through the study without realizing the potential for introducing a confound. Data collection should be monitored extremely carefully throughout the study to ensure that data acquisition is conducted as planned and head motion is minimized. Lastly, interpretations of these findings can be problematic because (a) the risk factors to which these infants were exposed are unparalleled in range and severity in middle‐ and high‐resource countries and (b) different risk factors may alter brain development differently (e.g. Sheridan & McLaughlin, 2014). These factors make comparisons of the present findings to MRI studies of adversity in higher income settings challenging. They also make it difficult to disentangle effects of individual risk factors.

### 4.2 Do biological and/or psychosocial risk factors explain amygdala/precuneus iFC?

It had been hypothesized that biological factors (e.g. malnutrition) emerging from severe financial hardship alter brain development (Jensen et al., 2017). Accordingly, these factors would mediate any observed relationships between income‐to‐needs and brain properties (Farah, 2017). Thus far, animal models have demonstrated that malnutrition is associated with brain development in terms of reductions in cortical cell size, density of cortical dendritic spines, myelin production, number of synapses, and number of glial cells (Keunen, Elburg, Bel, & Benders, 2015; Levitsky & Strupp, 1995); infections have been shown to damage white matter tracts via upregulation of pro‐inflammatory cytokines (Duggan et al., 2001; Hansen‐Pupp et al., 2005). Results of the present study do not demonstrate that the relationship between income‐to‐needs and amygdala/precuneus iFC at 2‐months‐of‐age is mediated by HAZ, which was employed as a proxy for malnutrition and exposure to infection. However, it is possible that HAZ mediates relationships between income‐to‐needs and measures of brain structure, such as brain volume, cortical thickness or functional anisotropy, either in specific areas/tracts or globally. As most infants from both groups were breastfed at this age, another possibility is that none were malnourished (yet); consequently, HAZ would not exhibit the variance that might emerge after greater exposure to malnourishment. It would be useful for subsequent studies with larger sample sizes to devise metrics that more closely approximate malnutrition and infection than HAZ alone.

Although the multiple regression results should be viewed as preliminary, they showed that prenatal family conflict explained iFC variance after controlling for income‐to‐needs and other risk factors. Importantly, the relationship between a form of early life stress and amygdala/parietal iFC is consistent with a study in 6‐month‐old infants in the U.S., which reported a positive correlation between interparental conflict (“a relatively moderate form of early life stress” that is similar to the family conflict measure in the present study) and amygdala/posterior cingulate cortex (PCC) iFC (Graham et al., 2015); notably, PCC and precuneus are adjacent structures and both are considered hubs in the default‐mode network (Utevsky, Smith, & Huettel, 2014). Mechanistically, the amygdala is tied to neuroendocrine responses through indirect projections to the HPA stress system (Gunnar & Quevedo, 2007) and glucocorticoid receptors (Wang et al., 2014), which regulate HPA output of glucocorticoids, but are dysregulatory following early life stress (Arnett et al., 2015). It is thought that stressful events drive the release of glucocorticoids, for which the amygdala has receptors (Wang et al., 2014), from the HPA system (Lupien et al., 2009). As with malnutrition, early life stress, as examined in rodents, is also thought to engender an inflammatory response. Although reports are inconsistent as to whether pro‐ or anti‐inflammatory effects are favored in the response to stress (Ganguly & Brenhouse, 2015), it should be noted that pro‐inflammatory cytokine concentrations in infants exhibited a negative association with later neurocognitive outcomes (Jiang et al., 2014, 2017).

Furthermore, the relationship between prenatal family conflict and amygdala iFC evinces an intermediate segment of a theoretical framework proposed by Noble and colleagues (2012) for children in which low SES drives stress, which drives structural and functional alterations in the amygdala. This may have downstream effects on various functional outcomes, since the amygdala is involved in mediating effects of early life stress on cognitive and emotional functioning in infancy (Graham et al., 2015) and later childhood and adolescence (Fareri & Tottenham, 2016; Tottenham & Galván, 2016). In this framework, SES comprises income‐to‐needs and education (Noble et al., 2012) and, in a subsequent iteration, also occupation, neighborhood, and subjective status (Brito & Noble, 2014). The results presented here do not support a mediation pathway by which income‐to‐needs specifically drives prenatal family conflict and prenatal family conflict drives amygdala alterations, because prenatal family conflict did not mediate the relationship between income‐to‐needs and amygdala/precuneus iFC. However, another type of stressor could mediate this relationship, as different forms of stress can have different and even opposing neurochemical effects; e.g. maternal separation during childhood is linked to upregulation of glucocorticoids, while abuse is linked to downregulation (for a review, please see Lupien et al., 2009). Future studies could examine whether the relationship between income‐to‐needs and amygdala/precuneus iFC is mediated by (a) the stable presence of the mother or other caregiver, which is thought to attenuate the response of the amygdala to stress by reducing secretion of glucocorticoids from the HPA system (Fareri & Tottenham, 2016)); (b) the same psychosocial variables as explored here but referencing the postnatal, rather than prenatal, time points; (c) sleep deprivation, which is associated with poverty (Patel, Grandner, Xie, Branas, & Gooneratne, 2010) and also relatively greater amygdala/precuneus iFC (Shao et al., 2014); or (d) environmental contaminants (Farah, 2017; Jensen et al., 2017). Lastly, it should be noted that the framework by Noble and colleagues (2012) was proposed in the context of older children (5–17 years‐of‐age in their study), not 2‐month‐old infants, who may be too young to embody maternal psychosocial stress. An alternate framework may be better suited to describing the effects of poverty on brain development at this age.

### 4.3 How early in life are poverty‐related iFC effects detectable?

The results of the present study demonstrate that poverty‐related iFC effects on the developing brain are detectable in the first two months of life. Although not tested directly, when considered in the context of previous literature, the cross‐sectional results here also speak to whether childhood‐poverty‐related iFC differences exhibited later in life are cumulative or occur during a sensitive period, when the brain is especially responsive to experiences (for a discussion of sensitive periods, please see Fox et al. (2010)). Specifically, Barch and colleagues (2016) observed poverty‐related iFC effects between amygdala and seven brain regions including precuneus in a sample of roughly 10‐year‐old children. Taken together with the amygdala/precuneus iFC finding in the present study, exposure to risk factors associated with poverty early in life might be sufficient to engender some, but not all, poverty‐related iFC alterations observed later in childhood. Future longitudinal studies (with larger sample sizes and improved data quality) will be needed to more thoroughly examine this question of timing.

### 4.4 Are poverty‐related iFC effects specific to certain brain areas?

The presence of a between‐group iFC effect for the amygdala, but not visual or auditory, seeds, suggests that risk factors associated with poverty may alter iFC in a regionally specific manner at this early point in life. Stated cautiously, this conclusion is bolstered by the convergence of previous literature on relationships between childhood income‐to‐needs and structures localized disproportionately to the medial temporal lobe (Barch et al., 2016; Javanbakht et al., 2015; Kim et al., 2013; Luby et al., 2013). Additionally, a resting‐state fMRI study in 6‐month‐old infants conducted in the U.S. found no significant association between income and visual, auditory, motor, or default‐mode network iFC after correcting for multiple comparisons (Gao et al., 2015). In contrast, infants in the present study were experiencing conditions of extreme poverty (Nelson, 2017), ostensibly with more varying degrees of sensory input (e.g. noise) than environments in the U.S; this would likely have presented a better opportunity to detect poverty‐related iFC effects, were any to exist in visual or auditory networks.

The approach taken in the selection of seed regions was based primarily on reported relationships between amygdala structure and function and childhood income‐to‐needs (Barch et al., 2016; Javanbakht et al., 2015; Kim et al., 2013; Luby et al., 2013). However, other brain regions may also exhibit vulnerability to factors associated with poverty. In particular, retrospective fMRI studies of adults with a history of childhood poverty have reported associations between childhood income‐to‐needs and brain function and/or functional connectivity in prefrontal cortex (Javanbakht et al., 2015; Kim et al., 2013; Sripada, Swain, Evans, Welsh, & Liberzon, 2014) and PCC (Sripada et al., 2014), in addition to the amygdala (Javanbakht et al., 2015; Kim et al., 2013; for a review, please see Pavlakis, Noble, Pavlakis, Ali, & Frank, 2015). Future resting‐state analyses of poverty in infants might consider examining networks other than those investigated in the present study; toward this end, graph‐theory approaches (e.g. Power, Fair, Schlaggar, & Petersen, 2010; van den Heuvel & Sporns, 2013) may be useful.

The location of these effects may also depend upon the risk factors investigated. In the present study, prenatal family conflict, a form of early life stress, was associated with amygdala alterations, which is consistent with this region's role in regulating stress (Doom & Gunnar, 2013). Other risk factors associated with poverty might engender alterations in other brain regions (Noble et al., 2012; Sheridan & McLaughlin, 2014).

Lastly, whether a brain region is vulnerable to the hazards associated with poverty may depend upon the region's growth and maturation rate at the time those hazards occur. The amygdala and sensory regions develop at different rates at 2‐months‐of‐age; e.g. amygdala volume increases rapidly during the first year of life (Uematsu et al., 2012), whereas primary sensory region volumes increase at substantially slower rates (Gilmore et al., 2012). Therefore, it is possible that alterations in amygdala iFC occur during this early sensitive period, whereas alterations in calcarine cortex and/or Heschl's gyrus iFC necessitate a longer, cumulative exposure to risk factors; this could be akin to persistent or recurrent auditory insult (e.g. loud noises) engendering tonotopic reorganization of auditory cortex (Elgoyhen, Langguth, De, & Vanneste, 2015; Yang, Weiner, Zhang, Cho, & Bao, 2011). Ultimately, detection of poverty‐related iFC effects in particular regions may also depend upon the timing and duration of exposure to biological and/or psychosocial hazards.

## 5 CONCLUSION

This is the first MRI study to examine brain function in infants growing up in Bangladesh, among the poorest countries in the world. Despite numerous unique challenges, we collected resting‐state fMRI data in 2‐month‐old Bangladeshi infants and confirmed that they exhibited resting‐state networks resembling those in high‐resource settings (Doria et al., 2010; Fransson et al., 2011, 2007; Gao et al., 2015, 2017; Qiu et al., 2015). Compared with infants from relatively higher income families, infants from extreme poverty exhibited greater (i.e. less negative) iFC in the amygdala, a brain area with structural and functional alterations that have previously been linked to income‐to‐needs in older populations (Barch et al., 2016; Javanbakht et al., 2015; Kim et al., 2013; Luby et al., 2013). Preliminary data‐driven analyses indicated that prenatal family conflict was also correlated with amygdala/precuneus iFC, highlighting the complex relationship among poverty, psychosocial adversity, and brain function in infants. The present work expands upon prior studies of poverty by examining infants exposed to extreme biological and psychosocial risk factors in the context of severe poverty. This pilot, along with neuroscientific findings from future studies with larger sample sizes and improved data quality, may inform policies that counteract the risk factors associated with global childhood poverty (Farah, 2018).

## DATA AVAILABILITY STATEMENT

The data that support the findings of this study are available from the corresponding author, Nadine Gaab, upon reasonable request.
